# Hodgkin lymphoma: the role of EBV plasma viral load testing in an HIV-endemic setting

**DOI:** 10.1007/s10238-024-01524-8

**Published:** 2024-11-26

**Authors:** J. Opie, Z. Mohamed, D. Chetty, J. Bailey, K. Brown, E. Verburgh, D. Hardie

**Affiliations:** 1 Department of Pathology, Division of Haematology, Groote Schuur Hospital, University of Cape Town and National Health Laboratory Service, Cape Town, South Africa; 2 Department of Radiation Oncology, University of Cape Town and Groote Schuur Hospital, Cape Town, South Africa; 3 Department of Pathology, Division of Anatomical Pathology, Groote Schuur Hospital, University of Cape Town and National Health Laboratory Service, Cape Town, South Africa; 4 Department of Medicine, Division of Clinical Haematology, University of Cape Town and Groote Schuur Hospital, Cape Town, South Africa; 5 Department of Pathology, Division of Virology, Groote Schuur Hospital, University of Cape Town and National Health Laboratory Service, Cape Town, South Africa

**Keywords:** Hodgkin lymphoma, EBV, HIV, Plasma EBV, DNA, Overall survival

## Abstract

South Africa has a high burden of human immune deficiency virus (HIV)-associated Hodgkin lymphoma (HL) which is typically Epstein–Barr virus (EBV) infected, detected by histological stains. Circulating plasma EBV derived from apoptotic EBV infected tumour cells is a potential biomarker. This study aimed to evaluate the role of plasma EBV load testing in newly diagnosed HL patients and correlate pretreatment plasma EBV levels, HIV status and EBV tumour status with overall survival (OS). Untreated HL patients were prospectively included. Polymerase chain reaction measured EBV plasma viral loads. Kaplan–Meier curves with log-rank tests estimated the impact of HIV, EBV tumour status and plasma EBV viral loads on OS. Multivariable analysis was performed using a Cox proportional hazards model. Receiver operative characteristic curve analysis determined cutoff plasma EBV DNA levels with optimal sensitivity, specificity and concordance with tumour EBV status. The 68 patients included 21 (31%) HIV +ve and 33 (49%) EBV tumour +ve. EBV plasma ≥ 10 000 IU/ml (*P* = 0.008), EBV +ve tumour (*P* = 0.014), HIV +ve status (*P* = 0.009) and age ≥ 45 years (*P* = 0.018) predicted poorer OS on univariate analysis. Plasma EBV levels > 762 IU/ml had 89.29% sensitivity and 96.77% specificity for detecting EBV +ve HL. High plasma EBV levels ≥ 10 000 IU/ml, HIV +ve status and EBV tumour +ve status predicted poorer OS. Plasma EBV levels > 762 IU/ml predicted EBV +ve tumour status with high sensitivity and specificity. Plasma EBV viral DNA testing is a promising biomarker for EBV +ve HL.

**Supplementary Information:**

The online version contains supplementary material available at 10.1007/s10238-024-01524-8.

## Introduction

In 2022 sub-Saharan Africa had the highest global burden of human immunodeficiency virus (HIV), and South Africa had more than 8 million people living with HIV (PLWH) [1]. Hodgkin lymphoma (HL) is a high-grade lymphoma of B cell origin, with increased incidence in PLWH [2–4]. Even in the era of antiretroviral therapy, the risk of HL in PLWH is 7.7-fold higher than in HIV negative (−ve) populations. HL has good therapeutic outcomes in well-resourced settings; however, in resource-restricted settings, outcomes are often poorer, and patients present with disseminated disease and bone marrow infiltration [5]. With the increase in the rollout of antiretroviral therapy in sub-Saharan Africa, the impact of HIV status on survival in HL has been less clear with some studies reporting that HIV +ve status does not negatively impact overall survival (OS) [6, 7].

The pathogenesis of HL in PLWH includes reduced immune surveillance, chronic antigen B cell stimulation and concomitant oncogenic viral infection with enhanced risk for virus-induced oncogenesis [8]. Epstein–Barr virus (EBV) is a ubiquitous virus which establishes lifelong latent infection in the host. EBV is known to immortalise human B lymphocytes in culture and may lead to EBV-associated lymphoma, particularly in the setting of immunodeficiency [9]. Hodgkin tumour cells are known to be EBV infected in 30–40% of HIV −ve HL patients; however, in PLWH, 95% or more HL cases are EBV infected which suggests cooperative oncogenesis between HIV and EBV. The gold standard for assessment of EBV status in tumour cells is by pathologist morphological review of EBV-encoded small RNA in situ hybridization (EBERish) or EBV-encoded latent membrane protein (LMP-1) stains of histological tissue [10].

Peripheral blood EBV DNA viral loads measured by real time polymerase chain reaction (RT-PCR) have been used as a non-invasive biomarker for EBV-associated lymphoma to measure EBV tumour status, tumour burden, prognosis and treatment response [11–14]. Plasma EBV DNA contains only cell free (cf) EBV, which is derived from apoptotic EBV infected tumour cells [15]. Whole blood includes latent EBV infected B lymphocytes, and therefore, healthy individuals may have low measurable whole blood EBV DNA levels potentially confounding interpretation [16]. EBV DNA loads in whole blood are higher in PLWH than in the general population and are restricted to the whole blood compartment which may increase the risk of EBV-associated malignancies [17]. Plasma EBV is therefore generally preferred to whole blood as a biomarker in EBV-associated malignancies [11, 13, 15, 18–20]. The role of EBV biomarkers in HIV-associated HL has not been clearly established, and hold promise in view of the high clinical burden of EBV-associated HL in HIV-endemic populations in sub-Saharan Africa [15, 21–25]. A technological challenge is that EBV viral load reporting is poorly standardised despite the development of international EBV DNA standards by the World Health Organization in 2016 [26].

Resource restricted settings typically have poor access to pathologists and the functional imaging required for staging and monitoring in HL [25, 27]. This study aimed to investigate the clinical utility of plasma EBV DNA viral load testing, and to evaluate the impact of EBV +ve tumour status and HIV in newly diagnosed HL. We aimed to establish cutoff plasma EBV DNA values with optimal sensitivity and specificity for EBV +ve tumour status, and to correlate the impact of high plasma EBV DNA levels ≥ 10 000 IU/ml, HIV +ve and EBV +ve tumour status on OS.

## Patients and methods

### Patients

Newly diagnosed HL patients presenting to Groote Schuur Hospital (GSH) were prospectively enrolled from 2019 to 2023. GSH is a large tertiary academic hospital in Cape Town, South Africa. Staging was performed according to the Lugano classification using positron-emission tomography combined with computed tomography (PET-CT), CT and/or bone marrow findings [28]. Clinical and laboratory data were extracted from medical and laboratory records. Information collected included age, sex, stage at diagnosis (I–IV), histological subtype, EBV tumour status by EBERish or LMP-1 stains, bone marrow infiltration by HL, coronavirus disease 2019 (COVID) status, HIV status, CD4 counts, antiretroviral therapy, chemotherapy received and survival (alive/dead) at 6 months and 24 months after diagnosis. Standard first-line chemotherapy was doxorubicin, bleomycin, vinblastine and dacarbazine (ABVD). Second-line therapy decisions were made on an individual patient basis at multidisciplinary team meetings. Patients found eligible for transplant were treated with high dose salvage chemotherapy regimens followed by autologous stem cell transplant if they achieved remission. The study was approved by the Human Research Ethics Committee (Number 376/2019) at the University of Cape Town.

### EBV DNA viral load testing

Peripheral blood was collected in EDTA prior to commencement of therapy. EBV DNA viral loads were measured using the Abbott 2000 Real Time system (Chicago, USA) on paired whole blood and plasma samples for each patient. This assay targets the *BLLF1* gene, which encodes the gp350/220 envelope glycoprotein of EBV. The lower quantifiable limit of the assay was 150 IU/ml. Samples with a detectable viral load < 150 IU/ml were assigned a value of 100 IU/ml for analytical purposes. Histopathological diagnoses of classical HL were made by qualified pathologists in laboratories accredited according to International Standard Organization (ISO) quality standards. Diagnostic formalin fixed paraffin embedded tissue blocks were stained with EBERish or LMP-1 to establish EBV infection status of Hodgkin tumour cells.

### Statistical analysis

Categorical variables were described using frequencies and percentages and compared using Pearson Chi-squared or Fisher’s exact tests. Numerical variables were described using medians and interquartile ranges (IQR) and compared using Wilcoxon rank-sum tests, as data were nonparametric. The Kaplan–Meier method was used to estimate OS which was defined as the time from date of diagnosis to date of death from any cause or date last seen (censored) at a public health facility in the Western Cape Province of South Africa. Kaplan–Meier curves were compared using log-rank tests to determine associations between predictor variables and OS (univariate analysis). Predictor variables with a *P* value < 0.2 in univariate analysis were considered most appropriate for multivariate analysis. A Cox proportional hazards model was used to assess the association between predictor variables and OS. Predictor variables with a *P* value < 0.2 in univariate analysis selected for multivariable analysis included age ≥ 45 years, HIV status and EBV plasma viral load > 10 000 IU/ml. Receiver operative characteristic curve (ROC) analysis was used to determine the cutoff value for plasma EBV DNA giving optimal sensitivity, specificity and concordance with tumour EBV status. Statistical analyses were performed using STATA version 18.0 (Stata corporation, College Station, Texas, USA), and 2-sided *P* values < 0.05 were considered statistically significant.

## Results

Baseline characteristics of the study population are presented in Table 1. The study enrolled 68 patients with a median age of 36 years [Interquartile range (IQR) 26–52 years]. 34 (51%) were female, and 21 (31%) were PLWH, of which 18 (86%) were receiving antiretroviral therapy. Tumours were EBV +ve in 33 (49%) of all HL patients; 20 (95%) PLWH and 13 (28%) of HIV −ve (*P* < 0.001). Overall, 54 (80%) presented with advanced disease, defined as Stage III or IV [28]. The most frequent histological subtype was nodular sclerosing in 37 (54%) of all HL patients; 6 (29%) of HIV +ve and 31 (66%) of HIV −ve patients. 7 (33%) of HIV +ve HL cases could not be histologically subclassified as they were diagnosed on bone marrow biopsy.

**Table 1:** Baseline characteristics of Hodgkin lymphoma patients

	Total cohort n = 68	HIV +ve n = 21 (31%)	HIV –ve n = 47 (69%)	*P* value
Female	34 (51%)	10 (48%)	24 (51%)	0.793
Median age (years)	36 (IQR 26–52)	39 (IQR 28–46)	33 (IQR 25–57)	0.841
PLWH receiving ART		18 (86%)		
CD4 count median (cells/ul) *		152 (IQR 105–286)		
<100		5 (25%)		
100-200		6 (30%)		
>200		9 (45%)		
EBV tumour positive**	33 (49%)	20 (95%)	13 (28%)	<0.001
Stage at diagnosis				0.552
I	1 (2%)	0	1 (2%)	
II	13 (19%)	4 (19%)	9 (19%)	
III	9 (13%)	1 (5%)	8 (17%)	
IV	45 (66%)	16 (76%)	29 (62%)	
Histological subtype				<0.001
Nodular sclerosing	37 (54%)	6 (29%)	31 (66%)	
Lymphocyte rich	12 (18%)	2 (10%)	10 (21%)	
Mixed cellularity	10 (15%)	5 (24%)	5 (11%)	
Lymphocyte depleted	1 (1%)	1 (5%)	0	
CHL unclassified***	8 (12%)	7 (33%)	1 (2%)	
Chemotherapy treatment				0.244
Died before treatment/no treatment	1 (1%)	1 (5%)	0	
First line only	52 (76%)	17 (81%)	35 (74%)	
First & second line	15 (22%)	3 (14%)	12 (26%)	
Survival at last follow up				0.037
Alive	56 (82%)	14 (67%)	42 (89%)	
Deceased	12 (18%)	7 (33%)	5 (11%)	

*ART* antiretroviral therapy; *CHL* classical Hodgkin lymphoma; *PLWH* people living with HIV; *IQR* interquartile range

*1 patient did not have a CD4 count available

**Detected by EBERish (for lymph node and tissue) and LMP-1 staining (for bone marrow biopsies)

***All CHL unclassified cases were diagnosed on bone marrow biopsy

One patient died before treatment. 52 (76%) patients received first-line chemotherapy only and 15 (22%) patients required second-line chemotherapy, with no significant difference in chemotherapy treatment according to HIV status (*P* = 0.244). There was no correlation between advanced disease at diagnosis and HIV +ve status (*P* = 1.000) nor EBV +ve tumour status (*P* = 0.282). Of the twelve HL patients in the cohort who died, four had documented COVID +ve tests in the 14 days prior to death. In two of these, their deaths were regarded as likely COVID related.

**Fig. 1:**
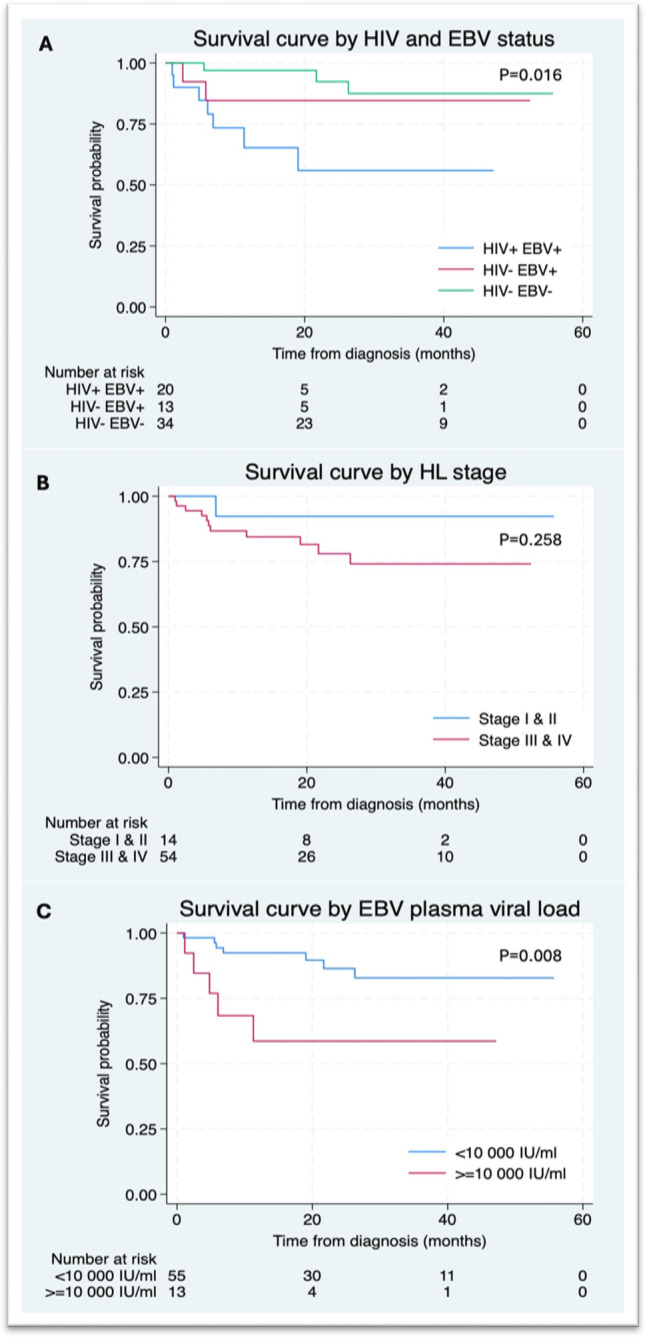
Kaplan–Meier survival curves in Hodgkin lymphoma patients

Kaplan–Meier survival curves are provided in Fig. 1. The total cohort survival probability was 91% [95% confidence interval (CI) 81–96%] at 6 months, and 81% (95% CI 68–89%) at 24 months. PLWH had poorer OS (*P* = 0.009), and a survival probability of 85.5% (95% CI 61.3–95.1%) at 6 months compared to 93.5% (95% CI 81.2–97.9%) for HIV −ve HL. The survival probabilities at 24 months were 58.9% (95% CI 30.9–78.7%) for PLWH compared to 89.6% (95% CI 73.6–96.2%) for HIV −ve HL patients. EBV tumour +ve status also correlated with significantly poorer survival (*P* = 0.014), and a 6-month survival probability of 84.53% (95% CI 66.71 -93.32%) compared to 97.1% (95% CI 80.94- 99.6%) for EBV −ve. The 24-month survival probability for EBV tumour +ve was 67.3% (95% CI 45.1–82.2%) versus 92.7% (95% CI 72.9–98.2%) for EBV tumour −ve patients. There was a significant difference between survival distributions when HIV and EBV tumour status were combined (Fig. 1A, *P* = 0.016). Specifically, EBV +ve PLWH had significantly poorer survival than those who were HIV −ve and EBV −ve (*P* = 0.004). No significant differences were noted for the other comparisons (HIV + EBV + vs. HIV − EBV +, *P* = 0.221 and HIV − EBV + vs. HIV – EBV−, *P* = 0.278). Comparing the survival by HIV and EBV tumour status, those that were both HIV+ and EBV tumour + did not show a significant difference in survival compared to HIV-EBV + (*p* = 0.221).

A high plasma EBV viral load level at diagnosis (≥ 10 000 IU/ml) was associated with a lower survival probability at 6 months of 76.9% (95% CI 44.2–91.9%) versus 89.4% (95% CI 63.8–97.3%) for plasma viral loads < 10 000 IU/ml. At 24 months the survival probability for those with EBV viral loads ≥ 10 000 IU/ml was 58.6% (95% CI 26.7–80.6%) compared to 73.4% (95% CI 40.7–89.9%) for those with a plasma EBV viral load < 10 000 IU/ml. Patients aged ≥ 45 years had a significantly shorter survival time than younger patients (*P* = 0.018). Patients with any detectable plasma EBV DNA also had a significantly shorter survival time compared to their EBV plasma DNA −ve counterparts (*P* = 0.021). There was no significant difference in OS for early-stage I and II versus advanced stage III and IV disease (*P* = 0.258) nor for males versus females (*P* = 0.777). Predictor variables selected for multivariable analysis included age, HIV status and EBV tumour status (Table 2). Age ≥ 45 years was associated with a poorer prognosis (hazard ratio (HR) 4.2, 95% CI 1.2–14.1) (*P* = 0.022). HIV +ve status (HR 3.2, 95% CI 1.0–10.3, *P* = 0.056) and a EBV plasma viral load ≥ 10 000 IU/ml (HR 3.4, 95% CI 1.0–11.5, *P* = 0.051) trended towards poorer OS.

**Table 2:** Multivariable analysis for the outcome death from any cause^*^

Covariate	Hazard ratio (95% CI)	*P* value
Age ≥ 45	4.2 (1.2–14.1)	0.022
HIV +ve	3.2 (1.0–10.3)	0.056
EBV plasma viral load ≥ 10 000 IU/ml	3.4 (1.0–11.5)	0.051

*Multivariable Cox proportional hazards model

**Fig. 2:**
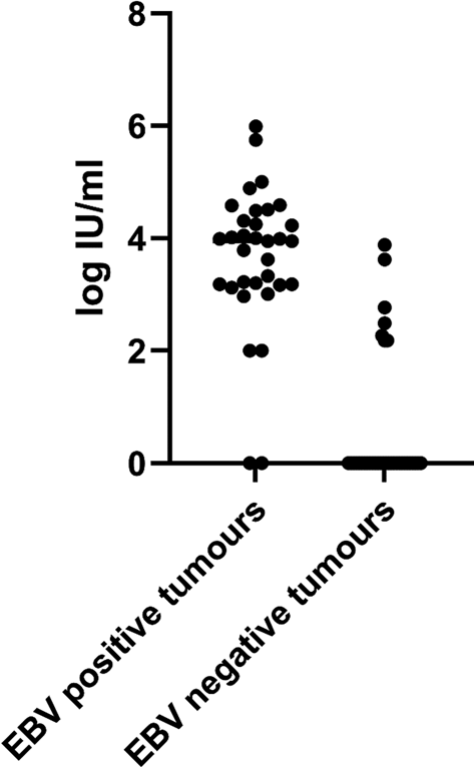
Plasma log EBV viral loads in Hodgkin lymphoma

Plasma EBV viral loads were significantly higher in EBV tumour +ve HL compared to EBV tumour −ve HL (*P* = 0.002) **(**Fig. 2). Median log viral loads in EBV +ve cases in whole blood and plasma were 4.010 IU/ml (IQR 3.465–4.530) and 3.950 IU/ml (IQR 3.175–4.40), respectively. In EBV tumour −ve cases, median log viral loads in whole blood and plasma were both 0 IU/ml with an IQR of 0–2.170 and 0–0, respectively. In patients with EBV +ve tumours, the EBV viral load values in paired whole blood and plasma samples were similar, differing by an average of 0.32 log IU/ml, with levels marginally higher in whole blood (supplementary Fig. 1).

Table 3 stratifies plasma EBV DNA viral loads in newly diagnosed HL according to EBV tumour status.

**Table 3:** Plasma EBV DNA viral loads in newly diagnosed Hodgkin lymphoma

EBV tumour status^#^	Positive	negative
Number of patients	*n* = 33	*n* = 35
EBV DNA plasma +ve^*^	*n* = 31/33 (94%)	*n* = 6/35 (17%)
Median viral load,	8 912	0
Range IU/ml	0–997 237	0–7 585
Viral load IU/ml IQR	1 496–25 118	0–0
Median Log IU/ml	3.950	0
Range IU/ml	0–5.990	0–3.880
Log viral load IQR	3.175–4.40	0–0

^#^Determined by EBERish or LMP-1 histological stains

*+ve defined as any detectable EBV DNA, IQR, interquartile range

31/33 (94%) EBV tumour + ve cases had detectable plasma EBV DNA. Two EBV tumour + ve cases tested plasma EBV −ve. The viral loads were repeated to confirm the findings in both cases. The first patient was a PLWH and advanced stage HL with bone marrow infiltration. The second patient was HIV −ve and had early-stage disease (stage II). Six EBV tumour −ve patients had detectable plasma EBV DNA. Using ROC curve analysis, a cutoff value of > 762 IU/ml EBV DNA plasma viral load had a sensitivity of 89.29% (CI 72.80–96.29) and specificity of 96.77% (CI 83.81–99.83) for detecting EBV +ve HL.

## Discussion

We found that the EBV plasma viral load was a valuable prognostic test with values ≥ 10 000 IU/ml associated with poorer survival (*P* = 0.008). HIV +ve status significantly correlated with poorer OS (*P* = 0.009) in univariate analysis and was associated with a 3.2-fold increased risk of death in multivariate analysis (*P* = 0.056). EBV tumour +ve HL patients had significantly poorer OS (*P* = 0.014) than EBV tumour −ve patients, which aligns with the findings of a large recent meta-analysis [29]. Our findings are noted to conflict with a recent local study which reported better survival in EBV tumour +ve HL, possibly due to less EBV +ve HIV −ve HL cases in their cohort [7]. We found age ≥ 45 years was associated with a 4.2-fold increased risk of death (*P* = 0.022) and high plasma EBV DNA viral loads ≥ 10 000 IU/ml with a 3.4-fold increased risk of death (*P* = 0.051). Our findings contrast with previous publications reporting no prognostic value for EBV DNA viral load testing in HIV-associated HL [21, 22] and support reports that high pretreatment levels of plasma EBV DNA are associated with inferior outcomes [11, 30].

Almost a third of this study cohort were PLWH, and 95% of them were EBV tumour +ve. Half our total cohort, 49%, were EBV tumour +ve, confirming the potential utility of an EBV biomarker for HL in our HIV-endemic setting. In view of limited resources in sub-Saharan Africa including pathologists and advanced imaging such as PET-CT scanning, this novel biomarker should be considered to assist with diagnosis, prognostication and monitoring of EBV-associated lymphomas. Using ROC curve analysis, a cutoff value of > 762 IU/ml plasma EBV DNA provided high sensitivity (89.29%) and specificity (96.77%) for EBV tumour +ve status. Future research directions include confirming these findings in larger patient cohorts and monitoring plasma EBV DNA viral load testing at treatment follow-up milestones and after completion of therapy to assess utility in our setting.

Most of our cohort, 79%, presented with advanced stage disease (Stage III and IV), irrespective of HIV status, which is known to negatively impact survival [31]. Unexpectedly, we did not find stage of disease to statistically correlate with survival, likely due to the small number of patients in the early-stage disease group. Total survival probability of the whole cohort was at 91% (CI 81–96%) at 6 months and 81% (CI 68- 89%) at 24 months, which is poorer than in well-resourced settings where there is a lower burden of HIV-associated HL, and more patients present with early-stage disease [32]. An important contributing factor to our patient’s late presentation is likely diagnostic delay due to the overlapping clinical symptoms and signs of tuberculosis (TB) and lymphoma. South Africa is a TB-endemic area, and diagnostic biopsies are difficult to access; thus, patients often receive empiric therapy for TB before an alternative diagnosis is considered or a biopsy obtained [33]. Furthermore, the COVID pandemic negatively impacted survival in our cohort, with several documented COVID-related deaths.

## Conclusion

This study assessed the prognostic role of pretreatment plasma EBV DNA viral loads, tumour EBV status and HIV status in HL patients in an HIV-endemic setting, where a high proportion of HL cases are EBV-associated. EBV tumour + ve status (*P* = 0.014), HIV + ve status (*P* = 0.009) and EBV DNA plasma levels ≥ 10 000 IU/ml (*P* = 0.008) were all significantly associated with poorer OS. A cutoff value of > 762 IU/ml EBV plasma DNA had high sensitivity and specificity for detecting EBV tumour + ve HL. EBV plasma DNA testing is a useful biomarker with potential to assist with early diagnosis and prognosis in EBV-associated HL.

## Supplementary Information

Below is the link to the electronic supplementary material.

Supplementary file 1 (DOCX 46 kb)

## Data Availability

The raw data are available in redcap and excel spreadsheets and can be provided if requested.
