# Correction: Hodgkin lymphoma: the role of EBV plasma viral load testing in an HIV-endemic setting

**DOI:** 10.1007/s10238-025-02025-y

**Published:** 2026-01-23

**Authors:** J. Opie, Z. Mohamed, D. Chetty, J. Bailey, K. Brown, E. Verburgh, D. Hardie

**Affiliations:** 1 Department of Pathology, Division of Haematology, Groote Schuur Hospital, University of Cape Town and National Health Laboratory Service, Cape Town, South Africa; 2 Department of Radiation Oncology, University of Cape Town and Groote Schuur Hospital, Cape Town, South Africa; 3 Department of Pathology, Division of Anatomical Pathology, Groote Schuur Hospital, University of Cape Town and National Health Laboratory Service, Cape Town, South Africa; 4 Department of Medicine, Division of Clinical Haematology, University of Cape Town and Groote Schuur Hospital, Cape Town, South Africa; 5 Department of Pathology, Division of Virology, Groote Schuur Hospital, University of Cape Town and National Health Laboratory Service, Cape Town, South Africa

**Correction to: Clinical and Experimental Medicine (2024) 25:10**

https://doi.org/10.1007/s10238-024–01524-8.

In the original version of the article, the entries in Table 1 was misaligned.

Table 1 which previously appeared as 

**Table:** Table 1. Baseline characteristics of Hodgkin lymphoma patients

	Total cohort n = 68	HIV+ ve n=21 (31%)	HIV- ve n=47 (69%)	P-value
Female	34 (51%)	10 (48%)	24 (51%)	0.793
Median age (years)	36 (IQR 26 – 52)	39 (IQR 28–46)	33 (IQR 25–57)	0.841
PLWH receiving ART		18 (86%)		
CD4 count median (cells/ul) * <100 100–200 >200		152 (IQR 105–286) 5 (25%) 6 (30%) 9 (45%)		
EBV tumour positive**	33 (49%)	20 (95%)	13 (28%)	<0.001
Stage at diagnosis I II III IV	1 (2%) 13 (19%) 9 (13%) 45 (66%)	0 4 (19%) 1 (5%) 16 (76%)	1 (2%) 9 (19%) 8 (17%) 29 (62%)	0.552
Histological subtype Nodular sclerosing Lymphocyte rich Mixed cellularity Lymphocyte depleted CHL unclassified***	37 (54%) 12 (18%) 10 (15%) 1 (1%) 8 (12%)	6 (29%) 2 (10%) 5 (24%) 1 (5%) 7 (33%)	31 (66%) 10 (21%) 5 (11%) 0 1 (2%)	<0.001
Chemotherapy treatment Died before treatment/no treatment First line only First & second line	1 (1%) 52 (76%) 15 (22%)	1 (5%) 17 (81%) 3 (14%)	0 35 (74%) 12 (26%)	0.244
Survival at last follow up Alive Deceased	56 (82%) 12 (18%)	14 (67%) 7 (33%)	42 (89%) 5 (11%)	0.037

ART antiretroviral therapy; CHL classical Hodgkin lymphoma; PLWH people living with HIV; IQR interquartile range*1 patient did not have a CD4 count available**Detected by EBERish (for lymph node and tissue) and LMP-1 staining (for bone marrow biopsies)***All CHL unclassified cases were diagnosed on bone marrow biopsy

But should have been

**Table:** Table 1. Baseline characteristics of Hodgkin lymphoma patients

	Total cohort n = 68	HIV+ ve n=21 (31%)	HIV- ve n=47 (69%)	P-value
Female	34 (51%)	10 (48%)	24 (51%)	0.793
Median age (years)	36 (IQR 26 – 52)	39 (IQR 28–46)	33 (IQR 25–57)	0.841
PLWH receiving ART		18 (86%)		
CD4 count median (cells/ul) * <100 100–200 >200		152 (IQR 105–286) 5 (25%) 6 (30%) 9 (45%)		
EBV tumour positive**	33 (49%)	20 (95%)	13 (28%)	<0.001
Stage at diagnosis I II III IV	1 (2%) 13 (19%) 9 (13%) 45 (66%)	0 4 (19%) 1 (5%) 16 (76%)	1 (2%) 9 (19%) 8 (17%) 29 (62%)	0.552
Histological subtype Nodular sclerosing Lymphocyte rich Mixed cellularity Lymphocyte depleted CHL unclassified***	37 (54%) 12 (18%) 10 (15%) 1 (1%) 8 (12%)	6 (29%) 2 (10%) 5 (24%) 1 (5%) 7 (33%)	31 (66%) 10 (21%) 5 (11%) 0 1 (2%)	<0.001
Chemotherapy treatment Died before treatment/no treatment First line only First & second line	1 (1%) 52 (76%) 15 (22%)	1 (5%) 17 (81%) 3 (14%)	0 35 (74%) 12 (26%)	0.244
Survival at last follow up Alive Deceased	56 (82%) 12 (18%)	14 (67%) 7 (33%)	42 (89%) 5 (11%)	0.037

The original version of this article has been corrected.

