# Supplementary material for: Hodgkin lymphoma: the role of EBV plasma viral load testing in an HIV-endemic setting
Source: Clin Exp Med. 2024 Nov 26;25(1):10. doi: 10.1007/s10238-024-01524-8 (PMC11599344; doi:10.1007/s10238-024-01524-8)
Supplement: Supplementary file 1 — (DOCX 46 kb) [file 10238_2024_1524_MOESM1_ESM.docx]

**Supplementary Figure 1:**

EBV DNA in paired plasma and whole blood samples of EBV tumour +ve Hodgkin lymphoma patients.


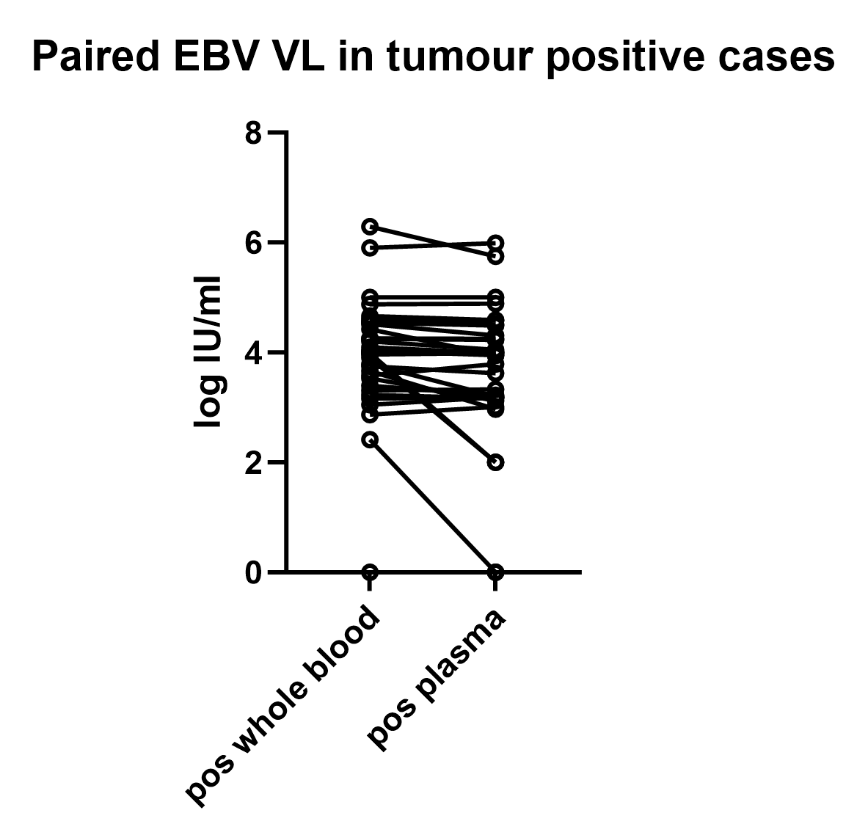


The scatter plot shows that EBV viral load in paired samples is very similar, with mean difference of 0.32 log IU/ml. EBV viral load in log IU/ml is indicated on the Y axis.
